# Common genetic variant association with altered HLA expression, synergy with pyrethroid exposure, and risk for Parkinson’s disease: an observational and case–control study

**DOI:** 10.1038/npjparkd.2015.2

**Published:** 2015-04-22

**Authors:** G T Kannarkat, D A Cook, J-K Lee, J Chang, J Chung, E Sandy, K C Paul, B Ritz, J Bronstein, S A Factor, J M Boss, M G Tansey

**Affiliations:** 1 Department of Physiology, Emory University School of Medicine, Atlanta, GA, USA; 2 Department of Epidemiology and Center for Occupational and Environmental Health, Fielding School of Public Health, University of California at Los Angeles, Los Angeles, CA, USA; 3 Department of Neurology, School of Medicine, University of California at Los Angeles, Los Angeles, CA, USA; 4 Department of Neurology and Movement Disorders Center, Emory University School of Medicine, Atlanta, GA, USA; 5 Department of Microbiology and Immunology, Emory University School of Medicine, Atlanta, GA, USA

## Abstract

**Background:**

The common noncoding single-nucleotide polymorphism (SNP) *rs3129882* in *HLA-DRA* is associated with risk for idiopathic Parkinson’s disease (PD). The location of the SNP in the major histocompatibility complex class II (MHC-II) locus implicates regulation of antigen presentation as a potential mechanism by which immune responses link genetic susceptibility to environmental factors in conferring lifetime risk for PD.

**Aims:**

The aim of this study was to determine the effect of this SNP on the MHC-II locus and its synergy with pesticide exposure.

**Methods:**

For immunophenotyping, blood cells from 81 subjects were analyzed by quantitative reverse transcription-PCR and flow cytometry. A case–control study was performed on a separate cohort of 962 subjects to determine association of pesticide exposure and the SNP with risk of PD.

**Results:**

Homozygosity for *G* at this SNP was associated with heightened baseline expression and inducibility of MHC class II molecules in B cells and monocytes from peripheral blood of healthy controls and PD patients. In addition, exposure to a commonly used class of insecticide, pyrethroids, synergized with the risk conferred by this SNP (odds ratio=2.48, *P*=0.007), thereby identifying a novel gene–environment interaction that promotes risk for PD via alterations in immune responses.

**Conclusions:**

In sum, these novel findings suggest that the MHC-II locus may increase susceptibility to PD through presentation of pathogenic, immunodominant antigens and/or a shift toward a more pro-inflammatory CD4+ T-cell response in response to specific environmental exposures, such as pyrethroid exposure through genetic or epigenetic mechanisms that modulate MHC-II gene expression.

## Introduction

The etiology of Parkinson’s disease (PD) remains largely unknown with <10% of cases attributable to an identifiable causative genetic mutation.^1^ The clinical diagnosis of PD by its hallmark motor symptoms may be preceded by various non-motor symptoms, including depression, anosmia, constipation, and random eye movement-sleep behavior abnormalities, some of which have been postulated to be fueled by inflammatory processes.^2,3^

Genetic polymorphisms in genes encoding glucocerebrosidase, α-synuclein, tau, and others have been reported to modify PD risk.^1^ Environmental exposures, such as pesticide exposure and head trauma are associated with increased risk for developing PD.^4,5^ Similar to other age-related diseases, current hypotheses suggest that genetic susceptibility must synergize with lifetime environmental exposures to initiate the development of PD pathology.^6,7^ The major histocompatibility complex class II (MHC-II) that is responsible for antigen presentation to the adaptive immune system may be particularly important in linking genetic background to environmental exposures.^8^ Inflammation has been implicated as a key driver of PD pathogenesis.^9^ Postmortem examination of PD brains has revealed microglial activation and lymphocyte infiltration in areas of degeneration.^10,11^ Increased expression of inflammatory cytokines, altered composition of peripheral immune cells, and the protective effects of chronic ibuprofen consumption further implicate inflammation in PD pathogenesis.^10,12,13^

The MHC-II locus contains the most highly polymorphic genes in the human population and mediates antigen presentation to CD4+ T cells and induction of adaptive immunity.^8,14^ MHC-II molecules present antigenic peptides on the surface of antigen-presenting cells (APCs), such as B cells, monocytes, macrophages, dendritic cells, and microglia.^8,14^ The MHC-II locus encodes three different α/β heterodimeric isotypes (HLA-DR, -DQ, and -DP).^8^ Each isotype has the potential to present distinct antigenic subsets to CD4+ T cells and induce their differentiation in a specified manner.^8^ Differentiated CD4+ T cells (Th1, Th2, Th17, and so on) promote specific inflammatory effector responses, or as regulatory T cells suppress inflammation.^14^ Given its key role in adaptive immunity, the MHC-II locus is an ideal candidate for linking the environment and genetic susceptibility to PD pathogenesis through inflammation.

Supporting a disease-promoting role for antigen presentation, multiple studies have identified associations between single-nucleotide polymorphisms (SNPs) in the MHC-II region and risk for late-onset PD.^15–24^ In several genome-wide association studies (GWAS), the *rs3192882* SNP has been associated with altered risk for PD;^15,16,25,26^ yet ethnic background appears to influence the allele associated with increased risk. In the largest GWAS to look at this SNP, homozygous carriers of the high-risk *G* allele (21% of PD patients and 16% of CTRLs) were found to have a 1.7-fold increased relative risk of developing PD in people of European ancestry.^15^ In addition, the *G* allele carried by 46% of PD patients and 40% of CTRLs was associated with increased levels of MHC-II as an expression-quantitative trait locus (eQTL) in subjects of European ancestry^19^ and more strongly associated with risk for sporadic PD rather than familial PD.^27^ As an eQTL, this SNP could be associated with genetic or epigenetic regulatory elements that modify the expression of the MHC-II locus. These data led us to hypothesize that the rs3129882 GG genotype is associated with increased surface and messenger RNA (mRNA) expression and greater inducibility of the MHC-II locus in peripheral immune cells relative to the AA genotype.

Given that the *rs3129882* SNP is located in the first intron of the monomorphic *HLA-DRA* gene and has not been associated with particular MHC-II haplotypes,^19^ it was somewhat surprising that a common genetic variant in an immune locus could influence the risk for a complex neurological disorder. Clearly, the genetic association between the MHC-II locus and risk for PD is complex and may depend on a variety of factors such as ethnic background, environmental exposures, and so on. As such, we hypothesized that this SNP would synergize with pesticide exposure, a known PD-relevant risk factor, to increase risk for PD, and this risk might be further modifiable by ethnicity and race. Given the heterogeneity of findings in various GWAS for MHC-II and risk for PD, the *rs3129882* SNP warranted further exploration as a possible genetic marker in certain populations associated with complex genetic and/or epigenetic mechanisms that modulate risk for PD by affecting antigen presentation.

## Materials and methods

### MHC-II expression cohort subject recruitment

PD patients and age-matched healthy CTRL subjects were recruited through the Clinical Research in Neurology Institutional Review Board-approved research protocol at the Emory Movement Disorders Clinic and community outreach events sponsored by the American Parkinson’s Disease Association, Wilkins Parkinson’s Foundation, and Emory Udall Center of Excellence for Parkinson’s Reasearch. Participants were excluded if they were younger than 50 years, were older than 85 years, or had neurological, chronic infectious, or autoimmune comorbidities, and/or known familial PD mutations. For subjects not originally in the Emory cohort of the Hamza *et al.* ^15^ study in which participants’ first HLA-DRA intron was sequenced, the *rs3129882* Taqman SNP Genotyping Assay (Life Technologies, Carlsbad, CA) was used to genotype newly recruited subjects. Subjects homozygous at the *rs3129882* locus were asked to provide a blood sample (~50 ml). At the time of recruitment, a questionnaire was used to assess disease and inflammation/immune-relevant environmental exposures and comorbidities. Caffeine, nonsteroidal anti-inflammatory drug, and nicotine intake was calculated as mg-years, dose-years, and mg-years, respectively. Levodopa equivalence dose was calculated based on parameters defined by the Parkinson’s Disease Society of the United Kingdom.

### PBMC isolation, sorting, and stimulation

PBMCs were isolated from whole blood using Ficoll-Paque (GE Healthcare, Atlanta, GA, USA) density centrifugation. The upper plasma layer was frozen immediately at −80 °C. B cells and monocytes were isolated by positive selection from total PBMCs using anti-CD19 and CD14 paramagnetic beads, respectively (Miltenyi Biotec, Bergisch Gladbach, Germany). Remaining cell fraction was analyzed by flow cytometry for quality control of sorting. For stimulation, monocytes were plated overnight with or without IFN-γ (PeproTech, Rocky Hill, NJ, USA) in a six-well plate at 5×10^5^ cells per well for flow cytometry or at 2×10^6^ cells per well for RNA isolation.

### RNA isolation, complementary DNA synthesis, and RT-PCR

Cells were washed once in ice-cold phosphate-buffered saline and then lysed in 350 μl RLT buffer (Qiagen, Venlo, The Netherlands) supplemented with 1% β-mercaptoethanol (Sigma-Aldrich, St Louis, MO, USA). Cell lysate was centrifuged through a Qiashredder (Qiagen) and then immediately frozen at −80 °C. Later, RNA was fully isolated using RNAeasy Isolation Kit (Qiagen) and stored at −80 °C. For complementary DNA synthesis, 0.5–2 μg of total mRNA was used per reaction in reverse transcription reactions using Superscript II (Life Technologies) with oligo dT and random hexamer primers (Life Technologies). The amount of SYBR-incorporated amplicons were measured for all real-time quantitative Bio-Rad iCycler instruments (Bio-Rad Laboratories, Hercules, CA, USA) with an iQ optical module were used to measure the amount of SYBR-incorporated amplicons for all real-time quantitative PCRs. DNA oligonucleotides (Integrated DNA Technologies, Coralville, IA, USA) used for primers listed in Supplementary Table S4 were diluted to a final concentration of 100 nM for PCRs. All primers were tested by agarose gel electrophoresis to ensure that they formed single-amplicon products of the correct size and optimized for Tm by temperature gradient real-time PCR followed by a melt curve analysis.

### Flow cytometry analysis

To stain for flow cytometry, 5×10^5^ cells per well were incubated in 1× FACS buffer (1% bovine serum albumin, 0.1% sodium azide, and 1 mM EDTA) for 20 min at 4 °C with anti-human HLA-DR:allophycocyanin/APC (1:20; BD Biosciences, San Jose, CA, USA, 559866), anti-human HLA-DQ FITC (1:20; BD Biosciences, 347453), anti-human CD14:phycoerythrin (1:100, Biolegend, no. 301806) and anti-human CD19:peridinin chlorophyll (1:100; Biolegend, no. 302228). Cells were fixed with 1% paraformaldehyde (Electron Microscopy Services, Hatfield, PA, USA) for 30 min at 4 °C. Cells were stored in 1× FACS buffer at 4 °C until run on FACS Calibur within 1 week of staining. Spherobeads (BD Biosciences) and OneComp Beads (eBiosciences) were used to set voltages and compensation settings between runs. Analysis of flow cytometry data was performed on FlowJo Software v10.0.6 (Ashland, OR, USA).

### Mesoscale discovery multiplex ELISA

Plasma was obtained from the upper layer of Ficoll-Paque separation and stored at −80 °C until sample analysis was performed. Plasma analyte levels were measured in duplicate using the Human Pro-inflammmatory Cytokine 7-plex, Human Chemokine V-PLEX, and Human C-reactive protein plates (Meso Scale Discovery, Rockville, MD, USA). For measurement of CRP, samples were diluted 1:200. For all other assays, samples were measured undiluted.

### Genevar analysis

Genevar 3.3.0 software (Wellcome Trust Sanger Institute, Hinxton, UK) was used to interrogate the HapMap3 cis-eQTL database. Association of levels of cis-eQTLs within 500 kb of the *rs3129882* SNP with SNP genotype for all the ethnic groups included in the database was reported as Spearman’s rank correlation (*ρ*) with *P* value.

### Pesticide exposure cohort and epidemiological methods

The Parkinson’s Environment and Gene (PEG) case–control study recruited incident PD cases and controls (CTRLs) from three highly agricultural counties in Central California, Kern, Fresno, and Tulare, between January 2001 and December 2013. Population-based CTRLs were recruited from the same tri-county study area and in the same age range as the cases using residential tax assessor’s records. All subjects were required to have lived in California for at least 5 years and PD patients were examined at least once by our movement disorder specialists, multiple times, and met published criteria for idiopathic PD.^28^ We described the details of case definitions^29^ and subject recruitment^30^ elsewhere. All procedures were approved by the University of California at Los Angeles Human Subjects Committee and informed consent was obtained from all participants.

In telephone interviews with patients and CTRLs, we obtained detailed information on demographic characteristics, risk factors, and lifetime occupational and residential histories. We estimated ambient pesticide exposures resulting primarily from commercial applications to agricultural crops using a geographic information system (GIS)-based computer model that links geocoded lifetime residential and occupations addresses of each participant to information on all commercial pesticide applications (date, location, and amount applied) from California State mandated pesticide use reports and land use data as published previously.^31^ For each pesticide, we summed the pounds applied per year per acre within a 500-m radius buffer of an address since 1974 (year of California State mandated pesticide use reports) and calculated study period average exposures for each subject and pesticide by summing the year-specific average exposures from 1974 to 10 years before the subject’s index year (date of diagnosis for patients and interview for CTRLs), and divided the sum by the number of years in the relevant time period. We substituted years missing a geocoded location with the average value of all recorded years for each pesticide and person. We then dichotomized exposures based on the pesticide-specific median level in exposed CTRLs (at or above). Participants could have received exposure at either residential or workplace addresses, or both.

The pesticide classes we examined were all previously identified as immunomodulatory.^32^ Aside from paraquat, each of the pesticides fell into the following three different chemical classes: organophosphates, dithiocarbamates, and pyrethroids. After assessing exposure to individual pesticides, we counted the total number of pesticides in each group that each participant was exposed to at each location (residence/workplace address), and classified each participant as highly exposed (at or above the median number of pesticides in exposed CTRLs) or receiving low/no exposure (none or below the median number of pesticides) based on the distribution for the total number of pesticides in exposed CTRLs, except for the pyrethroids, where 75% of the exposed CTRL population was only exposed to one pyrethroid pesticide, and we thus used the categories ‘no’ exposure versus exposure to one or more pyrethroid.

We used Student’s two-tailed *t*-test or a *χ* ^2^-test to investigate differences in demographic factors between patients and CTRLs. We assessed Hardy–Weinberg equilibrium for HLA *rs3129882* in CTRL participants using a *χ* ^2^-test, and then evaluated association between the SNP and PD using logistic regression to calculate ORs and 95% CIs, assuming a recessive and additive genetic model as done in prior reports,^15,26^ and adjusted for potential confounders including age (continuous, at diagnosis for patients and interview for CTRLs), sex, and ever/never smoking status (having smoked at least one cigarette per week for at least a year). To assess gene–environment interactions between HLA *rs3129882* and pesticide groups, we introduced a multiplicative interaction term into a logistic model assuming an additive genetic model and comparing only the *AA* and *GG* genotypes. Thus, we performed targeted analyses for specific chemicals (three different chemical classes and paraquat) selected *a priori* based on literature that suggest possible immune-modulatory effects of pesticides implicated in PD pathogenesis and for only one SNP. We used Quanto version 1.2.4^33^ for power calculations and SAS 9.3 (SAS Institute, Cary, NC, USA) for all other analysis. In order to determine the *rs3129882* genotype of the PEG cohort, samples were coded to blind the researchers and the *rs3129882* Taqman SNP Genotyping Assay (Life Technologies) was used for genotyping.

### Statistical analyses

A one-tailed Students’ *t*-test was used to make comparisons between the high-risk and low-risk genotype groups in the immunophenotyping studies at Emory University. One-way and two-way analysis of variance followed by Holm–Sidak *post hoc* tests were used when to compare the baseline characteristics of the study population and inducibility of the response to IFN-γ between the high risk and low risk, respectively, as indicated in figure legends. The Pearson coefficient was used to assess the correlation between two variables. GraphPad (Prism, La Jolla, CA, USA) and R (www.r-project.org; GNU project) software was used to perform statistical analyses. See “Pesticide Exposure Cohort and Epidemiological Methods” section above for detailed description of statistical methods used to interrogate the pesticide exposure cohort.

### Study approval

Written informed consent was received from participants before inclusion in the study. All participants provided written informed consent before inclusion in the research studies, and all procedures were approved *a priori* by the Institutional Review Board of Emory University in Atlanta, Georgia or of University of California at Los Angeles in Los Angeles, California.

## Results

### MHC-II expression study population

To assess the effect of *rs3129882* on MHC-II expression, 81 homozygous non-PD CTRL and PD subjects were recruited into the following four groups: CTRL *AA* (*n*=25), CTRL *GG* (*n*=12), PD *AA* (*n*=15), and PD *GG* (*n*=29). This strategy allowed us to examine the effect of SNP genotype, as well as disease status, on MHC-II expression. The groups of this study population were balanced with respect to factors that modify PD risk,^34–36^ including age, smoking, nonsteroidal anti-inflammatory drug use, caffeine intake, mean Unified Parkinson’s Disease Rating Scale motor score, levodopa equivalence dose, and duration of disease for the PD groups (Supplementary Table S1).

### The rs3129882 GG genotype is associated with increased surface MHC-II expression

**Figure 1:**
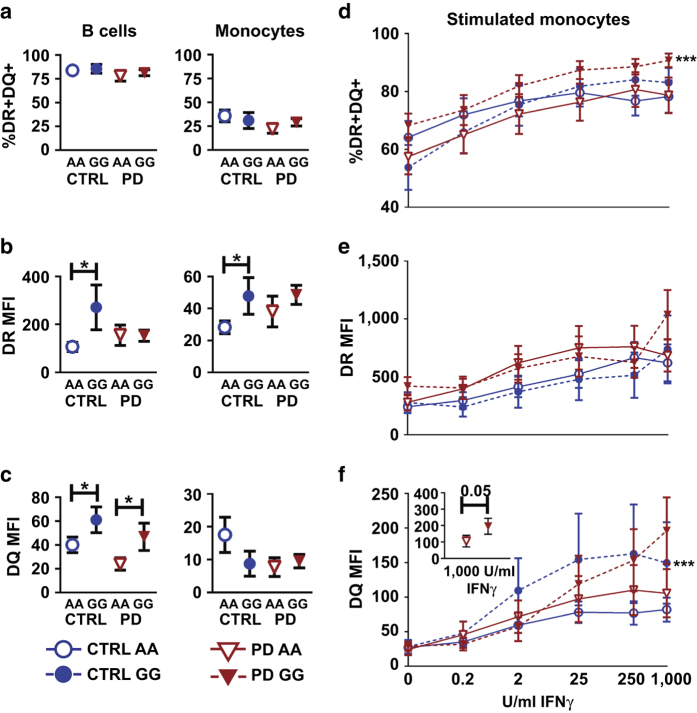
The high-risk rs3129882 GG genotype is associated with an increased level of major histocompatibility complex class II (MHC-II) expression in B cells and monocytes and with increased inducibility of surface HLA-DQ expression. (**a**) Frequency of HLA-DR/DQ double positive, (**b**) average level of HLA-DR expression, and (**c**) average level of HLA-DQ expression in B cells and monocytes was determined by flow cytometry staining of total peripheral blood mononuclear cells. One-tailed Student’s *t*-test between high-risk and low-risk allele groups was used to test for significance. *P<0.05. HLA-DR median fluorescence intensity (MFI): CTRL AA versus CTRL GG B cells *t*(33)=2.28, *P*<0.05; monocytes *t*(34)=2.14, *P*<0.05. HLA-DQ MFI CTRL AA versus CTRL GG B cells *t*(28) =1.76, *P*<0.05; PD AA versus PD GG *t*(35)=1.82, *P*<0.05. Surface MHC-II expression in paramagnetically, positively sorted monocytes stimulated with various concentrations of interferon-γ (IFN-γ) was measured by flow cytometry staining to measure increase in (**d**) frequency of HLA-DR/DQ double-positive cells, (**e**) level of HLA-DR expression, and (**f**) level of HLA-DQ expression. Two-way analysis of variance was performed to test for significance between GG and AA groups. ***P<0.001. (**d**) PD GG versus PD AA F(1,190)=11.97, *P*<0.001. (**f**) CTRL GG versus CTRL AA F(1, 163)=10.39, *P*<0.001. Inset for DQ MFI (**f**) panel shows sorted monocytes from PD patients stimulated with 1,000 U/ml IFN-γ. One-tailed *t*-test was performed for significance. *t*(31)=1.52, *P*=0.05. PD, Parkinson’s disease.

Given that the *rs3129882 G* SNP was associated with increased levels of MHC-II eQTL in subjects of European ancestry,^19^ we tested the hypothesis that in people of European ancestry, homozygosity for the *G* SNP would be associated with higher MHC-II expression compared with homozygosity for the *A* SNP. Flow cytometry was used to measure the frequency of cells expressing both HLA-DR and -DQ (DR/DQ double-positive cells) on the cell surface of APCs. We focused our analyses on B cells and monocytes because they are the major APCs in the peripheral blood. Nearly, all peripheral B cells and monocytes were HLA-DR positive (Supplementary Figure S1). In our cohort, 80% of peripheral blood B cells were HLA-DR/DQ double positive in all four groups of subjects (Figure 1a). Similarly, nearly all monocytes were HLA-DR positive but only 20–30% of peripheral blood monocytes were HLA-DR/DQ double positive in all four groups of subjects (Figure 1a). Using flow cytometry, the average level of HLA-DR or HLA-DQ surface expression was also measured, reported as median fluorescence intensity. In both B cells and monocytes from the CTRL *GG* group, there was a significant twofold increase in the median fluorescence intensity of HLA-DR compared to the CTRL *AA* group (Figure 1b). The median fluorescence intensity of HLA-DQ on B cells from *GG* individuals was also significantly increased in both CTRL and PD patients by 1.5- to 2-fold (Figure 1c). In this cohort, having the *GG* genotype was associated with increased median fluorescence intensity of HLA-DR on both B cells and monocytes, and of HLA-DQ on B cells.

### The rs3129882 GG genotype is associated with increased interferon-γ inducibility of HLA-DQ expression

Given that MHC-II expression increases upon cellular activation,^8,37^ we also measured disease- and genotype-specific effects on induction of this locus using interferon-γ (IFN-γ), a potent stimulator of MHC-II gene expression.^37,38^ In response to IFN-γ, the frequency of monocytes that became HLA-DR/DQ double-positive cells was significantly higher in the PD *GG* group relative to the PD *AA* group (Figure 1d). The level of induction of HLA-DR surface expression on monocytes was the same in all four groups of subjects (Figure 1e). In contrast, the HLA-DQ surface expression was significantly increased on IFN-γ-stimulated monocytes from CTRL *GG* individuals compared with CTRL *AA* individuals (Figure 1f). In PD *GG* patients, HLA-DQ surface expression was significantly increased relative to PD *AA* patients at the highest dose of IFN-γ (Figure 1f inset). In summary, the *rs3129882 GG* genotype was associated with increased HLA-DQ expression on monocytes in response to IFN-γ.

### The rs3129882 GG genotype is associated with increased baseline expression and IFN-γ inducibility of MHC-II mRNA

**Figure 2:**
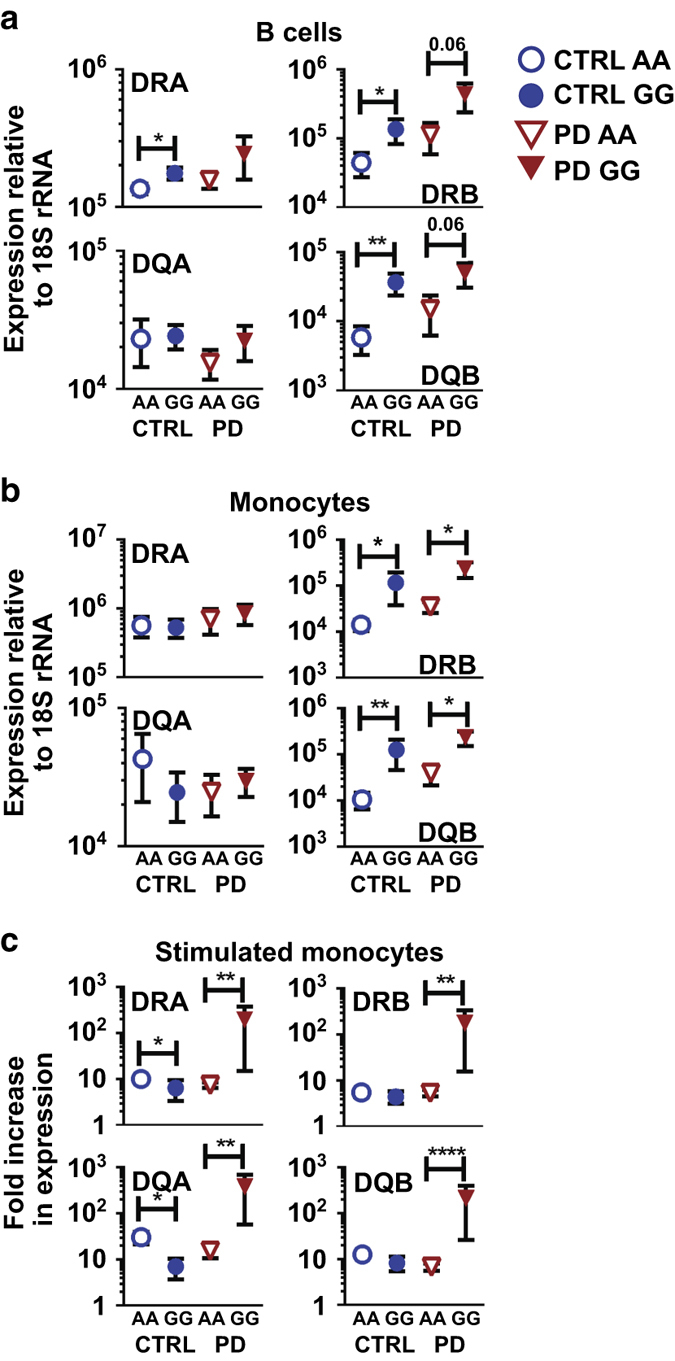
The high-risk rs3129882 GG genotype is associated with increased baseline expression and inducibility of major histocompatibility complex class II (MHC-II) messenger RNA (mRNA). RNA was isolated from paramagnetically, positively sorted (**a**) B cells and (**b**) monocytes. MHC-II mRNA expression was quantified relative to 18S rRNA with RT-PCR.*P<0.05, **P<0.01, ****P<0.0001. B-cell CTRL GG versus CTRL AA *HLA-DRA t*(35)=2.01, *P*<0.05; *HLA-DRB1 t*(34)=2.04, *P*<0.05; *HLA-DQB1 t*(33)=3.28, *P*<0.01. B-cell PD *GG* versus PD *AA HLA-DRB1 t*(25)=1.59, *P*=0.06; *HLA-DQB1 t*(33)=1.64, *P*=0.06. Monocytes CTRL *GG* versus CTRL *AA HLA-DRB1 t*(32) =1.90, *P*<0.05; *HLA-DQB1 t*(35)=2.08, *P*<0.01; PD *GG* versus PD *AA HLA-DRB1 t*(26)=2.24, *P*<0.05; *HLA-DQB1 t*(30)=2.28, *P*<0.05. (**c**) Fold change in MHC-II expression with or without 100 U/ml interferon-γ stimulation in paramagnetically, positively sorted monocytes was measured by RT-PCR after normalization to 18S rRNA levels. One-tailed *t*-test was performed as indicated. ****P<0.0001 **P<0.01, *P<0.05. CTRL AA versus GG *HLA-DRA t*(33)=1.77 *P*<0.05; *HLA-DQA1 t*(13)=1.80, *P*<0.05; PD AA versus PD GG *HLA-DRA t*(36)=1.76, *P*<0.01; *HLA-DRB1 t*(29)=1.65, *P*<0.01; *HLA-DQA1 t*(24)=1.82, *P*<0.01; *HLA-DQB1 t*(30)=2.53, *P*<0.0001. PD, Parkinson’s disease.

Next, we interrogated transcriptional levels of MHC-II to determine possible mechanisms underlying the association of *rs3129882* with altered surface MHC-II expression. To assess the extent to which APCs from *AA* versus *GG* individuals expressed differences in mRNA levels of the various MHC-II isotypes, we performed quantitative RT-PCR for the following four MHC-II genes in closest proximity to *rs3129882*: *HLA-DRA, -DRB1*, *-DQA1*, and *-DQB1* that are common among all MHC-II haplotypes. In B cells from CTRL subjects, the high-risk genotype was significantly associated with increased mRNA levels of *HLA-DRA*, *-DRB1,* and *-DQB1* (Figure 2a). In monocytes, the *GG* genotype was associated with increased *HLA-DQB1* mRNA expression in both PD patients and CTRLs, and with increased *HLA-DRB1* mRNA expression in PD subjects (Figure 2b). In addition to these statistically significant 10- to 20-fold increases in mRNA expression, there were clear upward trends in expression of *HLA-DRB1* and *-DQB1* mRNA in B cells from PD *GG* patients and *HLA-DRB1* mRNA expression in monocytes from CTRL *GG* subjects.

Following IFN-γ stimulation, monocytes from PD *GG* patients displayed a >200-fold increase in mRNA levels of all measured MHC-II genes relative to PD *AA* patients and CTRLs of either genotype (Figure 2c). With the exception of significantly decreased inducibility of *HLA-DQA* mRNA in the CTRL *GG* group, the inducibility of the rest of the MHC-II genes was the same in PD *AA* patients and CTRLs of either genotype. In summary, the *GG* genotype was associated with significantly increased *HLA-DRA, -DRB1*, and *-DQB1* mRNA expression in resting B cells independent of disease. In resting monocytes, the high-risk genotype was also associated with increased *HLA-DRB1* and *HLA-DQB1* mRNA expression independent of disease. Finally, monocytes treated with IFN-γ from the PD *GG* group displayed higher levels of all MHC-II mRNA.

### The rs3129882 high-risk genotype is associated with increased plasma CCL-3 (MIP-1α) levels in PD patients but not with altered frequencies of B cells and monocytes in the peripheral blood

In conjunction with cell-specific markers, differences in peripheral blood mononuclear cell (PBMC) composition and blood levels of cytokines/chemokines can indicate an active inflammatory process. Flow cytometry analysis of Ficoll-Paque-separated PBMCs demonstrated no change in B-cell or monocyte frequency between any of the study groups (Supplementary Figure S2A). The levels of 17 selected immunomodulatory cytokines and chemokines were measured by multiplexed chemiluminescent immunoassays (Supplementary Figure S2B). Individuals in the PD *GG* group displayed increased circulating plasma levels of CCL-3 (also known as MIP-1α) greater than twofold over the PD *AA* group. The levels of the other 16 cytokines and chemokines were not significantly different between the four groups (Supplementary Figure S2B).

### Pyrethroid exposure and the high-risk rs3129882 genotype increases odds for PD

To determine whether there are interactions between pesticide exposure and *rs3129882* in PD, 465 incident PD patients (diagnosed within 3 years of recruitment) and 497 population CTRLs of European ancestry from the PEG case–control study were examined. Basic demographic characteristics for the PEG study population can be found in Supplementary Table S2. As expected, patients were more likely to be male and have a family history of PD, and less likely to have ever been smokers.^2,36,39^ Our CTRL population was in Hardy–Weinberg equilibrium for HLA *rs3129882* (*P*=0.18). Genotype alone did not significantly influence PD risk, assuming previously reported genetic models (additive model: odds ratio (OR)=1.03, 95% confidence interval (CI)=0.86, 1.24 for those with one risk allele (*AG*) and OR=1.07, 95% CI=0.74, 1.55 for those with two risk alleles (*GG*); recessive model: OR=0.83, 95% CI=0.59, 1.16; Table 1).^15,26^ We did not detect interactions with organophosphates, dithiocarbamates, or paraquat and *rs3129882*.

Investigating pyrethroid pesticides, in logistic regression models, we estimated a positive interaction on a multiplicative scale when comparing groups homozygous at the SNP (*AA* versus *GG*; interaction *P* value=0.02; Table 2), and when we used an additive genetic model to include heterozygous individuals (interaction *P* value=0.007; Table 2). In both models, neither the genotype alone nor pyrethroid exposure alone significantly influenced PD risk, but in those jointly exposed to pyrethroids and having a *GG* genotype we estimated significant increases in PD risk. For example, when comparing *AA* versus *GG* in those unexposed, we see a slight nonsignificant decrease in PD risk (OR=0.73; 95% CI=0.47, 1.14), and no effect when comparing pyrethroid exposure in those with the *AA* genotype (OR=1.04; 95% CI=0.65, 1.67), yet comparing those homozygous for the risk allele (*GG*) and exposed to pyrethroids to those with the *AA* genotype and unexposed, we see an increased risk of PD (OR=2.48; 95% CI=1.24, 4.97; Table 2).

Next, we assessed whether there were differences in disease characteristics of the *AA* versus *GG* individuals at baseline and during two follow-up exams in a subset of the PEG population followed over time; mean years between baseline and exam 1 was 3.5 years (s.e.m.=0.1) and exam 2 was 5.6 years (s.e.m.=0.2). We did not see any significant differences in measures of disease-related clinical progression between *AA* and *GG* individuals at baseline or either follow-up time point (Supplementary Table S3).

### Genetic variation associated with ethnicity can reverse allelic rs3129882 association of MHC-II expression changes

In order to contextualize the discrepancies among various GWAS with associations of the *rs312988*2 with risk for PD, we used the GeneVar software tool (Hinxton, UK) to interrogate the HapMap3 cis-eQTL database. The HapMap3 database is unique in that it consists of 726 lymphoblastoid cell lines developed from individuals of eight different ethnic groups. We tested the hypothesis that association of SNP genotype with cis-eQTL level in the MHC-II locus would be affected by ethnicity. Indeed, we observed that in the individuals of the HapMap3 database, the allele that was associated with increased eQTL level depended on ethnicity (Table 3). For example, the *G* allele was associated with increased levels of *HLA-DRB1* eQTL in Utah Caucasians (*ρ*=0.199, *P*=0.038) but with decreased levels in Han Chinese (*ρ*=−0.281, *P*=0.0124) and Nigerian Yoruba (*ρ*=−0.317, *P*=8.0E−4). Furthermore, *HLA-DRB5* eQTL level was positively associated with the *G* allele in Utah Caucasians (*ρ*=0.516, *P*=9.3E−9) but negatively associated with the *G* allele in most of the other ethnic groups.

## Discussion

The MHC-II locus, and particularly the *rs3129882* SNP, has been implicated in modulating risk for PD;^15,16,21^ and herein, we demonstrate that the *G* allele of this SNP, acting together with environmental pyrethroids exposure, increases the odds of developing PD and is associated with altered MHC-II expression in peripheral APCs. The association of the high-risk genotype with altered MHC-II expression was revealed by: (1) increased surface protein expression of HLA-DR in monocytes; (2) greater inducibility of HLA-DQ surface protein expression in monocytes in response to IFN-γ; (3) increased mRNA expression of *HLA-DRA, -DRB1,* and *-DQB1* genes; and (4) greater inducibility of mRNA expression in PD patients’ monocytes after IFN-γ stimulation. Notably, within our MHC-II expression study cohort, more PD patients tended to be male and CTRLs, who were often caregivers, tended to be women. Despite this unequal sex distribution, stratification of the data by sex did not account for statistical differences in MHC-II expression (data not shown). Taken together, the data indicate that in our cohort, *GG* homozygosity at the *rs3129882* SNP is associated with increased baseline and inducible MHC-II expression in APCs. This immune hyper-responsiveness may in turn be one reason why this SNP has been associated with altering risk for late-onset PD. Specifically, the findings revealed that monocytes from individuals in our cohort with the *G* allele have more antigen-presenting capacity under resting conditions; and that within the context of PD, cytokine challenge increases mRNA expression of MHC-II genes ~200-fold. Surprisingly, plasma levels of cytokines were unchanged among all four groups except for increased MIP-1α in the PD GG group relative to the PD AA group, suggesting that immune risk for PD may be better predicted by cell-associated immune molecules rather than measurement of global fluid biomarkers. The pathological significance of an isolated elevation in a single plasma chemokine is unclear. These novel findings directly implicate regulation of MHC-II expression and antigen presentation as important mechanisms underlying the reported association between late-onset PD and the MHC-II locus. This evidence supports a role for immunological processes and the synergy between these processes and environmental exposures such as pyrethroids in determining an individual’s susceptibility to PD.

On the basis of the studies reported here, we speculate that the *rs3129882* SNP is a marker in some populations for a genetic and/or epigenetic mechanism(s) that control the expression of the MHC-II locus. A common mechanism in the MHC-II locus that promotes risk for PD may exist in many different human populations but may be marked by different disease-associated SNPs. Evidence for this is indicated by the divergent associations reported in GWAS in Han Chinese cohorts where the *A* allele is associated with increased PD risk, whereas in European cohorts the *G* allele is associated with increased risk.^15,16,25,26^ The ethnicity-dependent directional changes in the HapMap3 database cis-eQTL associations in Table 3 also point to the importance of taking into account ethnic differences when studying genetic variation at the MHC-II locus. Thus, the association of various MHC-II SNPs with PD likely depends heavily on ethnic makeup and exposome.

The expression of genes at this locus is regulated by a complex interplay between transcription factors, chromatin architecture, DNA methylation sites, and histone remodeling.^40^ In unstimulated cells from *GG* individuals in our cohort, mRNA expression of *HLA-DRA, -DRB1,* and *-DQB1* is increased, suggesting a mechanism that allows for basal increases in transcription of these genes (Figure 2a,b). The increase in the levels of β-subunit mRNAs is particularly interesting because the α-subunit mRNAs are typically much more abundant, making the level of β-subunit mRNAs an important limiting factor in production of mature MHC-II molecules. However, only in PD patients was the *rs3129882 G* allele associated with increased IFN-γ inducibility of mRNA expression of all the MHC-II genes. In CTRLs, the IFN-γ inducibility of *HLA-DQA* and *HLA-DRA* mRNA expression was significantly decreased (Figure 2c). This phenomenon suggests that in CTRLs, a regulatory mechanism may exist to limit the level of mRNA expression of MHC-II, i.e., a “ceiling effect” upon APC activation. If this turns out to be the case, our data suggest that this regulatory mechanism may be absent (or perhaps is lost as a result of the disease process itself) in individuals with a SNP in the MHC-II locus that hence increases risk of PD. Irrespective of the mechanism, enhanced transcription of MHC-II genes would be expected to increase surface expression of these molecules, resulting in functional consequences for engagement of the adaptive immune system.

Engagement of the adaptive immune system through MHC-II peptide presentation would allow for a specific, chronic inflammatory response mediated by CD4+ T cells.^14^ Shifts in the overall levels of MHC-II and/or the relative levels of MHC-II isotypes could impact normal immunological processes in two main ways. First, antigenic peptide epitopes that promote pathogenesis leading to PD may be more likely to be presented to the adaptive immune system and dominate immune responses.^41^ This could occur through skewed expression of HLA-DQ proteins. Second, the presentation of certain peptides on different MHC-II isotypes could alter the differentiation of CD4+ T cells into various subsets (i.e., Th1, Th2, Th17, or regulatory T cells).^42^ Evidence for such phenomena can be demonstrated in human autoimmune disease and humanized rodent models of autoimmunity.^43^ In this manner, antigen presentation via MHC-II on the surface of APCs to CD4+ T cells is a mechanism that could link the contribution of both genetic background and environmental exposure to susceptibility for developing PD.

**Figure 3:**
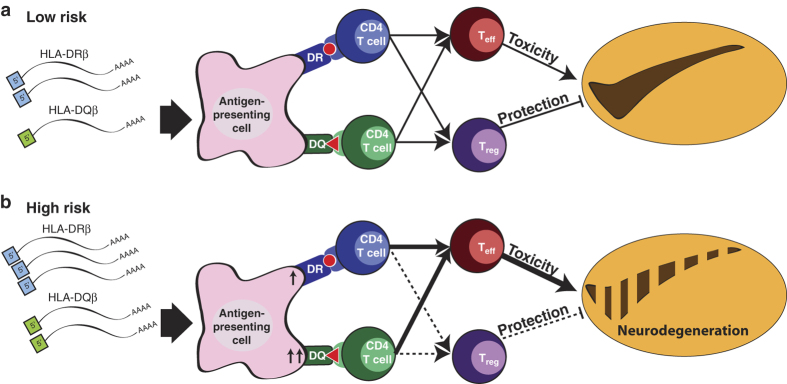
Model depicting the association of the rs3129882 single-nucleotide polymorphism (SNP) with altered major histocompatibility complex class II (MHC-II) expression on antigen-presenting cells and the potential for skewing the adaptive immune response and the predicted effects on vulnerable neuronal populations affected in Parkinson’s disease (PD). Our data suggest that the rs3129882 SNP is linked to a genetic or epigenetic element that increases messenger RNA expression of MHC-II and ultimately surface expression of MHC-II. Increased surface expression of MHC-II can influence CD4+ T-cell activation and differentiation leading to a heightened proinflammatory state that hastens neuronal dysfunction and death and predisposed individuals to PD. Two individuals exposed to the same environmental stimulus, one with the low-risk SNP (**a**) and the other with the high-risk SNP (**b**), are likely to respond differently immunologically because of underlying genetic and epigenetic mechanism(s) that influence MHC-II expression.

In the context of risk for PD, two people who are exposed to the same insult are likely to respond very differently immunologically given their genetic and epigenetic background. Within the context of our findings, following exposure to an environmental agent such as pyrethroids or an event such as traumatic brain injury, we would predict that an individual with the *AA* genotype in our cohort (Figure 3a) might display an immune response that resolves completely in a few weeks. By contrast, an individual from our cohort with the *GG* genotype (Figure 3b) might display a heightened immune response that may not resolve quickly but may instead promote chronic neuroinflammation, thereby hastening degeneration of vulnerable neuronal populations. Indeed, an adaptive immune response that is propagated in response to highly specific antigens differentially presented by APCs from people with high-risk versus low-risk MHC-II alleles can explain the selective degeneration of vulnerable neuronal populations as is the case in PD.

In agreement with our understanding of risk for PD, environmental stimuli, such as pyrethroids, are likely to have a prominent role in synergizing with the differential immune responses associated with this SNP given that few HLA haplotype associations have been reported for PD risk and none segregate with this SNP.^19^ Pyrethroid exposure has been associated in humans with acute alterations in immunoglobulin levels and T-cell frequencies^44^ and in animal models with altered APC function.^45^ Related to PD, pyrethroids are associated with increased striatal dopamine uptake, increased striatal dopamine metabolism, and altered electroencephalographic activity in the substantia nigra in chronically exposed rodent models.^46–48^ Other pesticides have been shown to directly impact antigen presentation, and although this has not been reported for pyrethroids it is possible that their effects on immune cells may explain the synergism with the MHC-II locus to increase risk for PD.^49^ The identified combined risk conferred by the *rs3129882 G* allele and pyrethroids may be explained by complex interactions that impact both the nervous system and the immune system to initiate PD pathogenesis through voltage-gated sodium channels that are known targets of this class of pesticides. The follow-up of the PEG patients-only cohort suggested no association between the *G* allele and disease severity/progression (Supplementary Table S3). However, longer follow-up time may be necessary to reveal differences in disease progression that are not yet apparent in this cohort. It is important to note that subject recruitment occurred through a state-wide registry with 30 years of exposure data; and PD patients underwent a neurological examination by movement disorder specialists. Our study is the first population-based analysis to explore and report pyrethroid–gene variant interactions, and thus it requires replication in an independent sample. Longitudinal studies of the MHC-II locus and PD would clarify the role of this locus in increasing risk.

The significance of our findings is threefold. First, we provided direct evidence from human peripheral blood that implicates a mechanism for antigen presentation and the role of adaptive immunity in risk for PD. Functional studies that link GWAS hits to functional cellular changes are the next step in understanding how genetics, environment, and cellular responses synergize to increase risk for complex diseases. Second, our findings suggest that the level and quality of MHC-II expression could prove to be an effective immune marker for the prediction of disease susceptibility in addition to honing our understanding of disease pathogenesis. Third, our data suggest that cellular biomarkers may prove more useful than soluble molecules in plasma and cerebrospinal fluid to identify individuals at risk for disease or for patient recruitment into neuroprotective trials testing immunomodulatory drugs.

## Figures and Tables

**Table 1:** HLA-DRA rs3129882 marginal effects in PEG population, *n*=962 (patients=465, controls=497).

*Genotype*	*Cases,* n *(%)*	*Controls,* n *(%)*	*Adjusted OR (95% CI)* a	P *value*
AA	142 (0.31)	175 (0.35)	1.00 (ref)	—
AG	243 (0.52)	227 (0.46)	1.03 (0.86, 1.24)	0.72
GG	80 (0.17)	95 (0.19)	1.07 (0.74, 1.55)	
AA versus GG:			0.95 (0.65, 1.38)	0.78
AA/AG versus GG			0.83 (0.59, 1.16)	0.27

Abbreviations: CI, confidence interval; OR, odds ratio; PD, Parkinson’s disease; PEG, Parkinson’s Environment and Gene.

An additive model was used to assess the association between the rs3129882 *G* allele and PD in the PEG population. Increased odds of developing PD was not associated with genotype alone in this population.

a Adjusted for age (continuous), sex, and smoking history.

**Table 2:** Interaction, main, and joint effect estimates between HLA rs3129882 and pyrethroid exposure in PEG study population of European ancestry, using both an additive genetic model and AA versus GG; *n*=962 (patients =465, controls =497)

*Ambient pyrethroids* a	*None*				*1+ Pesticide*			
	*Cases,* n *(%)*	*Controls,* n *(%)*	*Adjusted OR* b *(95% CI)*	P *value*	*Cases,* n *(%)*	*Controls,* n *(%)*	*Adjusted OR* b *(95% CI)*	P *value*
*Additive genetic model*								
* AA*	95 (0.30)	117 (0.33)	1.00 (ref)		47 (0.32)	58 (0.42)	0.83 (0.53, 1.28)	0.42
* AG*	172 (0.54)	161 (0.45)	0.91 (0.73, 1.13)	0.38	71 (0.48)	66 (0.48)	1.25 (0.88, 1.78)	0.22
* GG*	50 (0.16)	81 (0.23)	0.82 (0.53, 1.27)		30 (0.20)	14 (0.10)	1.87 (1.08, 3.35)	0.03
*P* value for interaction								*0.02*
*AA* versus *GG*								
*AA*	95 (0.66)	117 (0.59)	1.00 (ref)		47 (0.61)	58 (0.81)	1.04 (0.65, 1.67)	0.87
*GG*	50 (0.34)	81 (0.41)	0.73 (0.47, 1.14)	0.17	30 (0.39)	14 (0.19)	2.48 (1.24, 4.97)	0.01
*P* value for interaction								*0.007*

Abbreviations: CI, confidence interval; OR, odds ratio; PD, Parkinson’s disease; PEG, Parkinson’s Environment and Gene.

Using both an additive genetic model and comparing only the homozygous groups, we assessed the association between pyrethroid exposure and the risk rs3129882 genotype in the risk for PD. The table indicates the adjusted OR and *P* values for the main and joint effects and the *P* value for interaction.

a Ambient pesticide exposure to any pyrethroids (at or above the median level seen in exposed controls) at both occupation and residence, from 1974 (year of California State mandated pesticide use reports implementation) to 10 years before diagnosis or interview. Pyrethroid group includes fenvalerate, permethrin, phenothrin, resmethrin, flucythrinate, cypermethrin, (S)-cypermethrin, tau-fluvalinate, fenpropathrin, lamda-cyhalothrin, bifenthrin, esfenvalerate, and tralomethrin; Cyfluthrin had no exposure in study population.

b Adjusted for age (continuous), sex, and smoking history.

**Table 3:** The direction of association of cis-eQTL level with the rs3129,882 genotype depends on ethnicity

*HLA-DRB1*	ρ	P *value*	*HLA-DRB5*	ρ	P *value*
Utah Caucasians	0.199	0.038	Utah Caucasians	0.516	9.30E-09
Han Chinese	−0.281	0.0124	Han Chinese	−0.038	0.7396
Gujarati Indians in Houston, TX, USA	−0.017	0.8774	Gujarati Indians in Houston, TX, USA	0.242	0.0283
Japanese in Tokyo, Japan	0.038	0.7364	Japanese in Tokyo, Japan	−0.131	0.2393
Luhya in Kenya	−0.347	0.0014	Luhya in Kenya	−0.315	0.0039
Mexicans in Los Angeles, CA, USA	0.052	0.7333	Mexicans in Los Angeles, CA, USA	0.193	0.2029
Masaii in Kenya	−0.338	5.20E−05	Masaii in Kenya	−0.037	0.666
Yoruba in Nigeria	−0.317	8.00E−04	Yoruba in Nigeria	−0.122	0.2079

Abbreviations: eQTL, expression-quantitative trait locus; SNP, single-nucleotide polymorphism.

The level of HLA-DRB1 and HLA-DRB5 cis-eQTLs were significantly associated with rs3129882 genotype and were within 500 kb of the rs3129882 SNP in the HapMap3 database. Association was reported as Spearman’s rank correlation (*ρ*) with *P* value for all the ethnic groups within the database.
