# Supplementary material for: Common genetic variant association with altered HLA expression, synergy with pyrethroid exposure, and risk for Parkinson’s disease: an observational and case–control study
Source: NPJ Parkinsons Dis. 2015 Apr 22;1:15002–. doi: 10.1038/npjparkd.2015.2 (PMC4853162; doi:10.1038/npjparkd.2015.2)
Supplement: Supplementary Figure Legends [file npjparkd20152-s1.doc]

**Figure S1. Gating Strategy for Flow Cytometry Analysis.** Cells were stained for flow cytometry and analyzed on a FACS Calibur after standardization and compensation with Sphero beads, OneComp beads, and Raji and THP-1 cell lines. Gates were placed based on staining with isotype control antibodies. Analysis was performed and plots were generated using FlowJo software.

**Fig S2. The rs3129882 high-risk genotype is associated with increased plasma CCL-3 levels in PD patients with the high-risk rs3129882 GG genotype but not with altered frequencies of B cells and monocytes in the peripheral blood.**

A) B cell and monocyte frequencies were determined by flow cytometry staining of total PBMCs. B) Mesoscale Discovery (MSD) Multi-Array immunoassay technology was used to measure plasma levels of the indicated cytokines and chemokines. Values are plotted as the mean ± SEM. Two-tailed student’s T-test was used to test for significance between high risk versus low risk allele. * p < 0.05. CCL-3 PD GG vs PD AA t(16) = 2.39, p < 0.05.
