# Supplementary figures and images for: Common genetic variant association with altered HLA expression, synergy with pyrethroid exposure, and risk for Parkinson’s disease: an observational and case–control study
Source: NPJ Parkinsons Dis. 2015 Apr 22;1:15002–. doi: 10.1038/npjparkd.2015.2 (PMC4853162; doi:10.1038/npjparkd.2015.2)

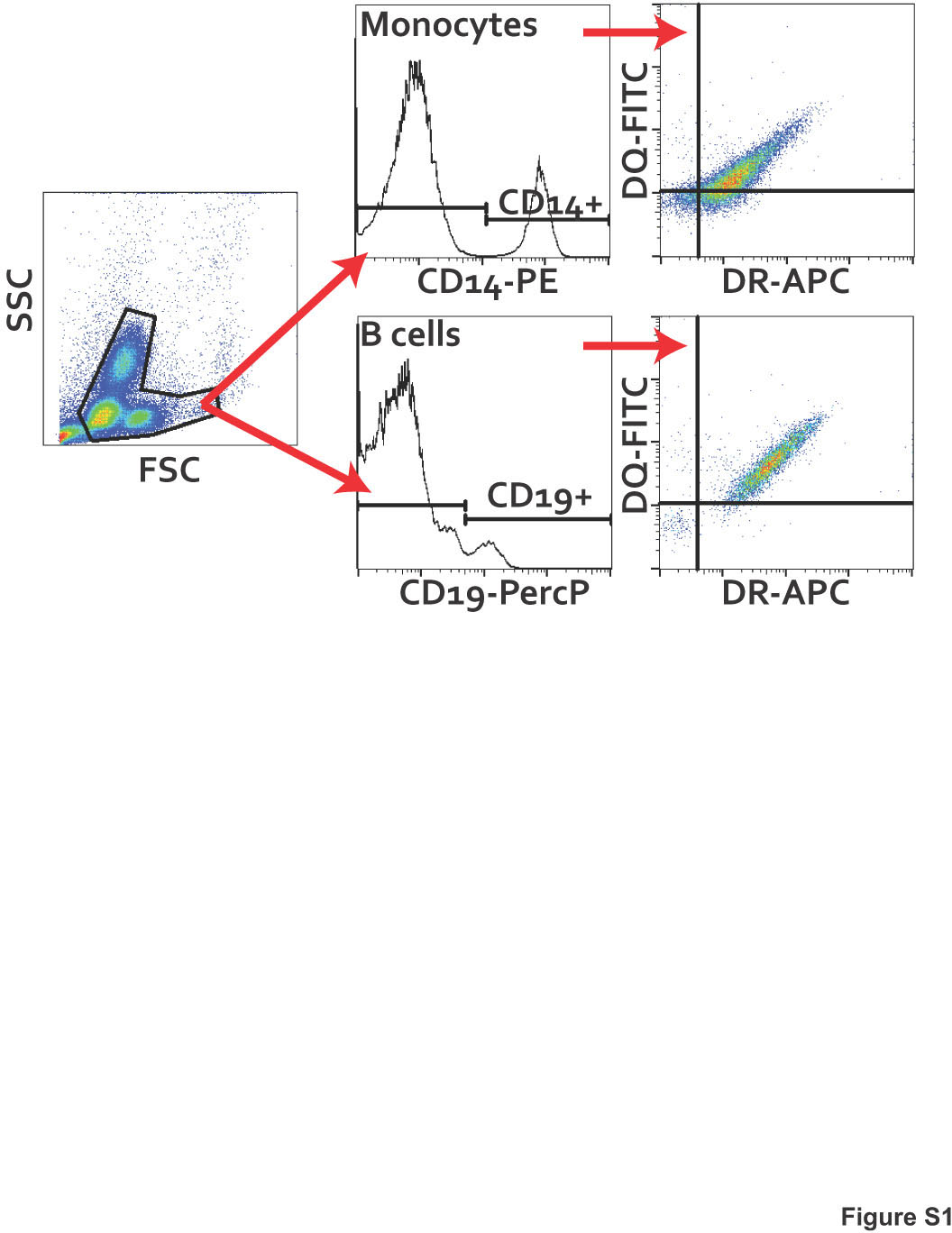

Supplement: Supplementary Figure S1 [file npjparkd20152-s2.jpg]

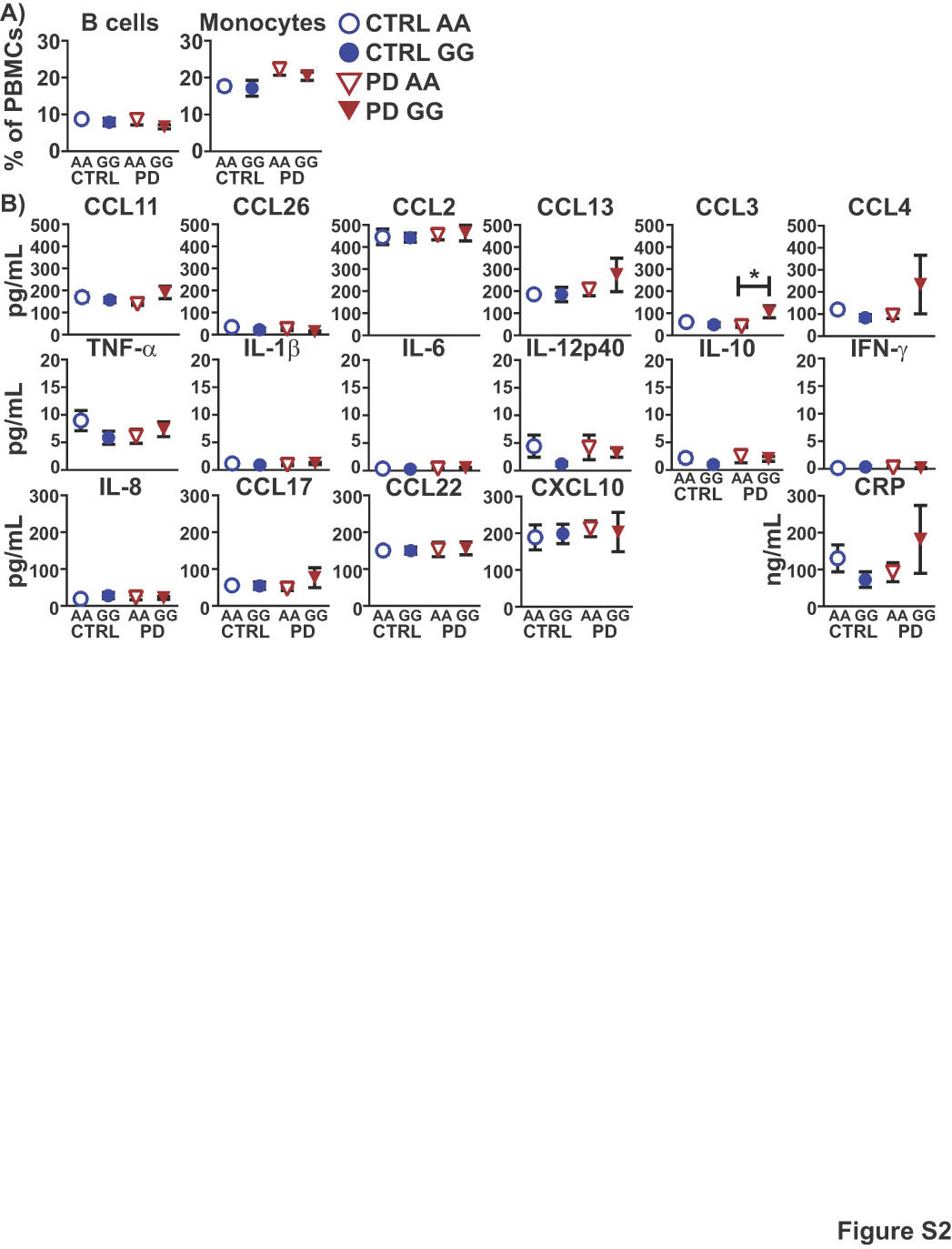

Supplement: Supplementary Figure S2 [file npjparkd20152-s3.jpg]
