# Supplementary material for: Common genetic variant association with altered HLA expression, synergy with pyrethroid exposure, and risk for Parkinson’s disease: an observational and case–control study
Source: NPJ Parkinsons Dis. 2015 Apr 22;1:15002–. doi: 10.1038/npjparkd.2015.2 (PMC4853162; doi:10.1038/npjparkd.2015.2)
Supplement: Supplementary Table S1 [file npjparkd20152-s4.doc]

**Table S1. Characteristics of MHC-II Expression Study Population.**

Data are expressed as the mean ± SEM for each of our four groups (CTRL AA, CTRL *GG*, PD *AA*, PD *GG*) and the entire study population. The table shows that our four groups are well balanced for the age, the self-reported environmental exposures, and clinician reported disease severity (UPDRS) measures. One-way ANOVA indicates no significance between groups at p > 0.05 for age, smoking, caffeine, NSAID use, number of lifetime head injuries, age at diagnosis, years with disease, mean UPDRS score, and levodopa equivalence dose. Chi-square test indicates significant difference (p = 0.003) in proportion of males to females in groups but stratification of data by sex does not explain statistical differences in MHC-II expression (data not shown).

|  | **CTRL AA** | **CTRL GG** | **PD AA** | **PD GG** | **All** | **p-value** |
| --- | --- | --- | --- | --- | --- | --- |
| **N** | 25 | 12 | 15 | 29 | 81 |  |
| **Age** | 65.5 ± 1.8 | 63.7 ± 1.8 | 67.4 ± 2.2 | 68.5 ± 1.5 | 66.6 ± 0.9 | 0.38 |
| **Sex** | 19F, 6M | 11F, 1M | 6F, 9M | 9F, 20M | 45F, 36M | 0.003 |
| **Smoking (pack-yrs)** | 2.9 ± 1.9 | 7.5 ± 3.2 | 8.3 ± 4.6 | 12.2 ± 5.0 | 7.8 ± 2.1 | 0.43 |
| **Caffeine**  **(mg-yrs)** | 6856 ± 1822 | 7959 ± 2068 | 5913 ± 1385 | 5907 ± 1341 | 6515 ± 806 | 0.49 |
| **NSAID use (dose-yrs)** | 1.95 ± 0.71 | 3.37 ± 1.3 | 4.34 ± 1.2 | 3.72 ± 1.0 | 3.30 ± 0.52 | 0.36 |
| **Number of Lifetime Head Injuries** | 0.22 ± 0.15 | 0.78 ± 0.36 | 0.47 ± 0.22 | 0.67 ± 0.26 | 0.50 ± 0.12 | 0.49 |
| **Age at Diagnosis** |  |  | 58.5 ± 3.0 | 59.5 ± 1.6 | 59.2 ± 1.5 | 0.40 |
| **Years with Disease** |  |  | 8.5 ± 2.0 | 9.0 ± 1.1 | 8.8 ± 0.95 | 0.78 |
| **Mean UPDRS Score** |  |  | 15.4 ± 2.9 | 16.8 ± 1.5 | 16.4 ± 1.4 | 0.41 |
| **Levodopa Equivalence Dose (mg)** |  |  | 222.5 ± 37.2 | 261.2 ± 45.5 | 244.4 ± 30.11 | 0.53 |
