# Supplementary material for: Common genetic variant association with altered HLA expression, synergy with pyrethroid exposure, and risk for Parkinson’s disease: an observational and case–control study
Source: NPJ Parkinsons Dis. 2015 Apr 22;1:15002–. doi: 10.1038/npjparkd.2015.2 (PMC4853162; doi:10.1038/npjparkd.2015.2)
Supplement: Supplementary Table S2 [file npjparkd20152-s5.doc]

**Table S2. General characteristics of PEG study population of European ancestry, n=962 (patients=465, controls=497).**

Data are expressed as the mean ± SEM for patients versus controls. The table shows that the PD patient group in the PEG study population is slightly older, more likely to have a family history of PD, has more males, and people with a history of cigarette smoking.

| **Characteristic** | **Patients (n=465)** | **Controls (n=497)** | **P-Value^a^** |
| --- | --- | --- | --- |
| **Age^b^, mean ± SEM** | 69.4 ± 0.46 | 67.4 ± 0.52 | 0.005 |
| **Age at PD onset, mean ± SEM** | 67.8 ± 0.53 |  |  |
| **Male sex, n (%)** | 289 (0.52) | 262 (0.48) | 0.003 |
| **Family history of PD^c^, n (%)** | 76 (0.16) | 47 (0.09) | 0.001 |
| **Ever cigarette smoker, n (%)** | 212 (0.46) | 271 (0.54) | 0.005 |
| **Years with Disease, mean ± SEM** | 2.49 ± 0.10 |  |  |
| **UPDRS-III Score^d^, mean ± SEM** | 21.0 ± 0.48 |  |  |
| ^a^P-values based on comparison between cases and controls using with chi-square or t-test | | | |
| ^b^Age at PD diagnosis for patients and interview for controls | | | |
| ^c^First degree relative with PD; 3 patients missing PD family history | | | |
| ^d^17 patients missing UPDRS-III score | | | |
