# Supplementary material for: Common genetic variant association with altered HLA expression, synergy with pyrethroid exposure, and risk for Parkinson’s disease: an observational and case–control study
Source: NPJ Parkinsons Dis. 2015 Apr 22;1:15002–. doi: 10.1038/npjparkd.2015.2 (PMC4853162; doi:10.1038/npjparkd.2015.2)
Supplement: Supplementary Table S3 [file npjparkd20152-s6.doc]

**Table S3. Clinical characteristics of PEG PD patients of European ancestry, across follow-up exams by HLA rs3129882 genotype (AA vs GG)**

Data for clinical characteristics presented as mean +/- SEM. Follow-up exam 1 was a mean of 3.5 yrs after baseline (SEM=0.1) and exam 2, 5.6 yrs after baseline (SEM=0.2). In this table, subjects homozygous for the rs3129882 SNP are compared in terms of clinical disease characteristics over time. There is no difference in the clinical characteristics between the groups at any time point.

|  | **Baseline with follow-up (n=88)** | | **Follow-up 1 (n=88)** | | **Follow-up 2 (n=69)** | |
| --- | --- | --- | --- | --- | --- | --- |
|  | AA (n=53) | GG (n=35) | AA (n=53) | GG (n=35) | AA (n=41) | GG (n=28) |
| Age at PD diagnosis | 65.3 ± 1.4 | 69.3 ± 1.6 | -- | -- | -- | -- |
| Age at PD onset | 63.7 ± 1.8 | 67.3 ± 2.1 | -- | -- | -- | -- |
| PD Duration (years) | 2.0 ± 0.2 | 1.6 ± 0.2 | 5.8 ± 0.4 | 5.1 ± 0.4 | 7.8 ± 0.5 | 7.2 ± 0.5 |
| Follow-up (years) | -- | -- | 3.8 ± 0.3 | 3.4 ± 0.3 | 5.8 ± 0.3 | 5.5 ± 0.4 |
| **Clinical features** |  |  |  |  |  |  |
| MMSE Score | 28.6 ± 0.2 | 28.5 ± 0.3 | 28.3 ± 0.3 | 28.2 ± 0.4 | 28.0 ± 0.4 | 27.4 ± 0.6 |
| GDS | 3.1 ± 0.5 | 2.4 ± 0.4 | 3.7 ± 0.4 | 3.7 ± 0.6 | 3.3 ± 0.5 | 3.4 ± 0.5 |
| UPDRS Score | 17.7 ± 1.0 | 17.0 ± 1.4 | 24.7 ± 1.4 | 23.7 ± 1.9 | 29.8 ± 1.7 | 27.2 ± 2.3 |
| *Resting tremor* | 1.4 ± 0.2 | 1.3 ± 0.2 | 2.1 ± 0.3 | 2.0 ± 0.4 | 2.2 ± 0.5 | 2.0 ± 0.4 |
| *Bradykinesia* | 5.9 ± 3.5 | 5.5 ± 3.9 | 7.8 ± 0.6 | 7.9 ± 0.9 | 9.9 ± 0.7 | 9.1 ± 1.0 |
| *Rigidity* | 2.7 ± 0.2 | 2.5 ± 0.4 | 3.4 ± 0.3 | 3.0 ± 0.4 | 4.9 ± 0.5 | 4.7 ± 0.7 |
| *Postural reflex impairment* | 2.6 ± 0.2 | 2.8 ± 0.3 | 3.7 ± 0.4 | 3.7 ± 0.4 | 5.4 ± 0.6 | 4.9 ± 0.6 |
| *p<0.05, based on genotype group comparisons using a t-test | | | | | | |
