# Supplementary material for: Common genetic variant association with altered HLA expression, synergy with pyrethroid exposure, and risk for Parkinson’s disease: an observational and case–control study
Source: NPJ Parkinsons Dis. 2015 Apr 22;1:15002–. doi: 10.1038/npjparkd.2015.2 (PMC4853162; doi:10.1038/npjparkd.2015.2)
Supplement: Supplementary Table S4 [file npjparkd20152-s7.doc]

**Table S4. RT-PCR Primers. List of primers used for qRT-PCR to measure mRNA expression of MHC-II genes.**

| **Target** | **Forward Primer (5’-3’)** | **Reverse Primer (5’-3’)** |
| --- | --- | --- |
| **HLA-DRA** | **GAGTTTGATGCTCCAAGCCCTCTCGC** | **CAGAGGCCCCCTGCGTTCTGCTGCA** |
| **HLA-DRB1** | **TGCTGAGCTCCCTACTGGCT** | **CGCGTACTCCTCTCGGTTATAG** |
| **HLA-DQA** | **CACCTTTTCTCTGGGACTTAAGC** | **TGAGGAATTAGGTAGCCGGGT** |
| **HLA-DQB** | **TATGCCTGCCCAGAATTCCC** | **CCATCAAGGCGGACCATGTGT** |
| **18S rRNA** | **GTAACCCGTTGAACCCCATT** | **CCATCCAATCGGTAGTAGCG** |
